# Pesticides and Organic Agriculture

**DOI:** 10.1289/ehp.112-a865b

**Published:** 2004-11

**Authors:** Katherine DiMatteo

**Affiliations:** Organic Trade Association, Greenfield, Massachusetts, E-mail: kdimatteo@ota.com

I read with horror the article “Pesticides and Parkinson Disease” by Renee Twombly (2004) in which she implied that rotenone is “often used in organic gardening and farming.” She went on to describe the effects of rotenone and the even more harmful effects of pyridaben, which is far more toxic than rotenone, both of which are used in conventional agriculture.

To set the record straight, rotenone is not commonly used in organic agriculture. Rotenone that has been naturally derived is listed as a “restricted substance” by the Organic Materials Review Institute (OMRI 2004) and may be used only in special circumstances with designated limitations. Meanwhile, rotenone’s synergist, piperonyl butoxide, is prohibited from use in organic agriculture.

The premise of organic agriculture is to fortify the soil through wholesome, nontoxic means, thereby strengthening the ability of plants to defy diseases and pests.

It is the hope of the hardworking pioneers in the organic movement that the instance of Parkinson disease, cancer, and many environmentally related illnesses will diminish exponentially with the conversion of acreage to organic cultivation.

*Editor’s response:* As DiMatteo implies, rotenone is, or should be, used only as a last resort in organic gardening and farming. It should be noted, however, that this pesticide is commonly marketed and sold under the rubric “organic gardening supplies.”
